# Vasoactive and/or inotropic drugs in initial resuscitation of burn injuries: A systematic review

**DOI:** 10.1111/aas.14095

**Published:** 2022-06-16

**Authors:** Kristine Knappskog, Nina Gjerde Andersen, Anne Berit Guttormsen, Henning Onarheim, Stian Kreken Almeland, Sigrid Beitland

**Affiliations:** ^1^ Department of Plastic, Hand and Reconstructive Surgery, Norwegian National Burn Center Haukeland University Hospital Bergen Norway; ^2^ Department of Anaesthesiology, Division of Emergencies and Critical Care Oslo University Hospital Oslo Norway; ^3^ Department of Anaesthesia and Intensive Care Haukeland University Hospital Bergen Norway; ^4^ Department of Clinical Medicine, Faculty of Medicine University of Bergen Bergen Norway; ^5^ Specialised Health Care services Quality and Clinical Pathways, Norwegian Directorate of Health Oslo Norway

**Keywords:** burns, cardiotonic agents, critical illness, fluid resuscitation, systematic review, vasoconstrictor agents

## Abstract

**Background:**

According to current guidelines, initial burn resuscitation should be performed with fluids alone. The aims of the study were to review the frequency of use of vasoactive and/or inotropic drugs in initial burn resuscitation, and assess the benefits and harms of adding such drugs to fluids.

**Methods:**

A systematic literature search was conducted in PubMed, Embase, Cochrane Database of Systematic Reviews, Cochrane Central Register of Controlled Trials, UpToDate, and SveMed+ through 3 December 2021. The search included studies on critically ill burn patients receiving vasoactive and/or inotropic drugs in addition to fluids within 48 h after burn injury.

**Results:**

The literature search identified 1058 unique publications that were screened for inclusion. After assessing 115 publications in full text, only two retrospective cohort studies were included. One study found that 16 out of 52 (31%) patients received vasopressor(s). Factors associated with vasopressor use were increasing age, burn depth, and % total body surface area (TBSA) burnt. Another study observed that 20 out of 111 (18%) patients received vasopressor(s). Vasopressor use was associated with increasing age, Baux score, and %TBSA burnt in addition to more frequent dialysis treatment and increased mortality. Study quality assessed by the Newcastle‐Ottawa quality assessment scale was considered good in one study, but uncertain due to limited description of methods in the other.

**Conclusion:**

This systematic review revealed that there is a lack of evidence regarding the benefits and harms of using vasoactive and/or inotropic drugs in addition to fluids during early resuscitation of patients with major burns.

**Editorial Comment**

The quantity and quality of studies on the resuscitation of burn‐injured patients are low. Given the concerns about fluid overload and adverse skin effects from vasoactive drugs, clinical trials are urgently warranted.

## 1 INTRODUCTION

Burn wounds are dynamic, and both depth and surface area may progress after the initial injury.^1^, ^2^ Optimal treatment may reverse vulnerable tissue surrounding the irreversibly damaged center of a burn injury.^1^ The direct tissue injury from the burn triggers a release of systemic inflammatory mediators, development of a strong negative interstitial pressure, loss of endothelial glycocalyx structure, increased capillary permeability, and rapid edema formation.^3^ This leads to the cardiovascular dysfunction known as the burn shock, and the resulting hypovolemia, hypotension, and/or hypoperfusion may become an urgent threat to life.^2^, ^4^, ^5^ Thus, the aim of initial fluid resuscitation of burn patients is to prevent the development of hypovolemic burn shock with as little fluid as possible.^5^

Initial fluid resuscitation with crystalloids is generally recommended in major burns covering more than 20% total body surface area (TBSA).^6^, ^7^, ^8^ Many different formulae have been suggested to predict fluid requirements in severe burns. The original Parkland, Evans, and Brook formulae are the most well‐known and cited.^9^, ^10^, ^11^ These formulae and their modified versions still remain in current practice guidelines.^6^, ^8^, ^12^ However, resuscitation of severe burns is complicated by the trans‐vascular fluid shifts leading to a rapidly developing burn oedema and the risk of over resuscitation.^13^ The resulting fluid overload can become life threatening due to complications such as muscular and/or abdominal compartment syndrome and/or compromised airways.^14^, ^15^, ^16^

Strategies to limit the amount of fluid by the use of colloids, sub‐target resuscitation, or the use of vasoactive and/or inotropic drugs have been proposed.^17^ However, current guidelines do not recommend the use of vasoactive and/or inotropic drugs in the initial resuscitation phase (0–24 h).^6^, ^8^, ^12^ The use of vasoactive and/or inotropic drugs may have beneficial effects but might also cause harm due to increased risk of cardiac arrhythmia and reduced perfusion of partially burnt skin.^18^

The aims of the study were to review the frequency of use of vasoactive and/or inotropic drugs in initial burn resuscitation, and to assess benefits and harms of adding such drugs to fluids.

## 2 MATERIALS AND METHODS

### 2.1 Purpose

The purpose of the present study was to perform a systematic review and meta‐analysis of initial resuscitation of burn patients admitted to the intensive care unit (ICU), to identify, evaluate, and summarize the findings of all relevant individual studies on use of vasoactive and/or inotropic drugs.

### 2.2 Aim

The aims were to review how often vasoactive and/or inotropic drugs (norepinephrine, epinephrine, dopamine, and/or dobutamine) were added to intravenous fluids during initial resuscitation of patients with major burns, and to assess benefits and harms of adding vasoactive and/or inotropic drugs. A PICO (population, intervention, comparison, outcome) diagram is presented in Table 1.

**TABLE 1:** PICO (population, intervention, comparison, outcome) diagram of research question

Population	Intervention	Comparison	Outcome
Burn injury patients admitted to the intensive care unit (ICU)	Use of intravenous fluids (any type and volume) combined with vasoactive and/or inotropic drugs (norepinephrine, epinephrine, dopamine and/or dobutamine) within the first 48 h after burn injury	Use of intravenous fluids (any type and volume) without vasoactive and/or inotropic drugs (norepinephrine, epinephrine, dopamine and/or dobutamine) within the first 48 h after burn injury	Use of vasoactive and/or inotropic drugs Use of intravenous fluids Risk factors for use of vasoactive and/or inotropic drugs Organ function parameters Antibiotic treatment Surgical procedures Mortality Length of stay Health care costs

### 2.3 Study registration and reporting

This systematic review was registered in the Prospero database 20 February 2019 (CRD42019120317).^18^ The study protocol with primary outcomes and statistical analyses was published before the study was conducted. Results were reported according to the Preferred Reporting Items for Systematic Reviews and Meta‐Analyses (PRISMA) guidelines and a PRISMA checklist is available in Appendix A.^19^

### 2.4 Search strategy

A systematic literature search was performed through 3 December 2021 in the following electronic databases to identify relevant studies on burns and the vasoactive/inotropic drugs dobutamine, dopamine, epinephrine, and norepinephrine: PubMed, Embase, Cochrane Database of Systematic Reviews, Cochrane Central Register of Controlled Trials, UpToDate and SveMed+. The search was conducted by an experienced librarian in collaboration with an intensive care clinician (SB) and was not limited by year of publication or type of publication. Studies published in English, Swedish, Danish, and Norwegian languages were considered. A detailed search strategy is attached in Appendix B.

### 2.5 Study selection

Two collaborators (KK and NGA) independently screened studies for eligibility according to predefined study selection criteria (Appendix C). Inclusion criteria were studies of burn patients admitted to the ICU comparing use of fluids alone with addition of vasoactive and/or inotropic drugs during initial resuscitation. Exclusion criteria were use of other study populations, use of vasoactive and/or inotropic drugs more than 48 h after burn injury and lack of a control group receiving intravenous fluid alone. Any disagreement was resolved through discussion with a senior author (SB). Both observational and interventional studies were considered for inclusion in order to summarize all available evidence.

### 2.6 Data extraction

Two independent collaborators (KK and NGA) extracted available data in duplicate according to a predefined data extraction form (Appendix D). This included data on study design, number of included patients, use of vasoactive and/or inotropic drugs, types of burn injuries, % TBSA burnt and presence of inhalation injury. Additional data were age, sex, risk factors for use of vasoactive and/or inotropic drugs, organ function parameters, and patient outcomes such as length of stay and mortality. Any disagreement was resolved through discussion with a senior author (SB).

### 2.7 Assessment of study quality

Two authors (KK and NGA) independently assessed the risk of bias of included studies using the Newcastle‐Ottawa quality assessment scale, which is a well‐established tool for assessing the quality of nonrandomized studies in meta‐analyses.^20^ Any disagreement was resolved through discussion with a senior author (SB).

### 2.8 Quantitative data synthesis

According to our protocol, we planned to perform a meta‐analysis using random effect models if the number of included studies was three or more, and to perform subgroup analyses covering the following topics: Burn injury population, burn injury mechanism, severity of burn injury and/or severity of organ failures.^18^

## 3 RESULTS

### 3.1 Study selection

**FIGURE 1:**
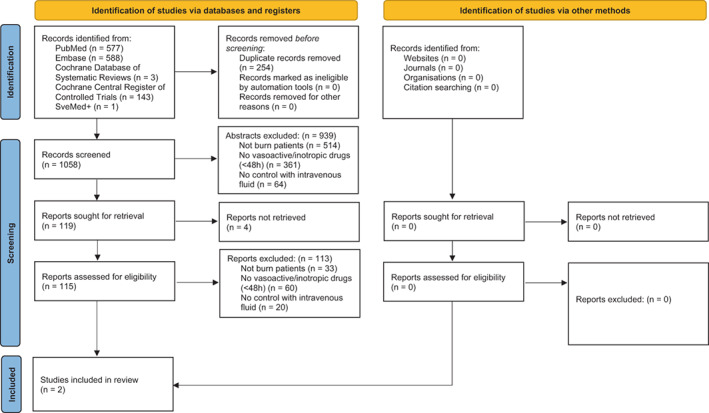
Preferred Reporting Items for Systematic Reviews and Meta‐Analyses (PRISMA)^19^ flow chart demonstrating how the publications identified through our systematic literature search were screened for eligibility.

The literature search identified 1058 unique publications that were screened for inclusion. After assessing 115 publications in full text, two retrospective cohort studies were included (PRISMA flowchart presented in Figure 1).^21^, ^22^ One of them was published as a conference abstract, the other as a full article. Among records not included, four were not available when sought from two separate medical libraries.

### 3.2 Data extraction

We extracted data from the two included studies by Pape et al and Adibfar et al.^21^, ^22^ The main findings are presented in Table 2. In these studies, vasoactive and/or inotropic drugs were used in 18% and 31% of the patients, respectively.

**TABLE 2:** Main characteristics and results of included studies

Reported data	Adibfar et al^22^	Pape et al^21^
Year published	2021	2019
Country	Canada	USA
Study design	Retrospective cohort study	Retrospective cohort study
Publication type	Article	Abstract form
Population	Adults with burns ≥ 20% TBSA	Adult burn injury patients
Inclusion period	November 15, 2015–July 30, 2018	Five‐year period (time unknown)
Study participants	Total number of patients: 52 Age: Not described (>16 years) TBSA burnt: Not described	Total number of patients: 111 Age: Described as adults TBSA burnt: Not described
Inclusion criteria	Adults with burn injury ≥ 20% TBSA, admitted within 24 h post burn	Adults burn injury patients treated with fluid resuscitation
Exclusion criteria	Patients revived from cardiac arrest with vasoactive drugs prior to BC arrival, administered continuous VP prior to BC arrival, palliative comfort measures ≤ 24 h post burn, age < 16 years	Patients dead within 24 h
Intervention group (VP+ group)	Number of patients: 16 Age: 55.3 years, 43.8% female TBSA burn: 44%; full thickness: 33.8% Mortality: 56% VP use: Norepinephrine, epinephrine, vasopressin, phenylephrine. Initiated 20.9 ± 10.9 h post burn, mean total duration of infusion 16.8 ± 10.8 h RF use: 5.7 ± 2.3 ml/kg/%TBSA at 24 h	Number of patients: 20 Age: 54.6 years, sex not described TBSA full thickness burn: 37.7% Mortality: 45% VP use: Drug type not described. Initiated 4.97 ± 11.2 h after admission and continued for 18.7 ± 45.9 h RF use: LR 15.9 L first 24 h
Control group (VP− group)	Number of patients: 36 Age: 42.3 ± 16 years, 22% female TBSA burn: 25%; full thickness: 14.5% Mortality: 11% RF use: 5 ± 1.8 ml/kg/%TBSA at 24 h	Number of patients: 91 Age: 42.2 years, sex not described TBSA full thickness burn: 14.5% Mortality: 17.6% RF use: LR 10.9 L first 24 h
Reported significant outcomes	Parameters significantly higher in VP+ group: • TBSA total and full thickness % burnt • Use of mechanical ventilation • In hospital mortality • Acute kidney injury • Administration of HDVC	Parameters significantly higher in VP+ group: • Age • TBSA full thickness % burnt • RF volume first 24 h • Baux score • Mortality rate • Dialysis requirement
Main results	Higher age, larger and deeper %TBSA burn, need of mechanical ventilation, and use of HDVC was associated with increased use of VP. Albumin administration was associated with reduced VP requirements	Older patients with higher Baux score and greater full‐thickness burns are more likely to require VP during acute fluid resuscitation. VP use was correlated with need for dialysis and mortality

Abbreviations: BC, burn center; HDVC, high dose vitamin C; LR, lactated ringer; RF, resuscitation fluid; TBSA, total body surface area; VP, vasopressor.

The Adibfar study included 52 patients in one group given vasopressor and fluids (PRESSOR group) (*n* = 16) and one receiving only fluids (NoPRESSOR group) (*n* = 36) during the first 48 h following burn injury.^22^ Patients in the PRESSOR group had a significantly higher need for mechanical ventilation compared to the NoPRESSOR group. Using multivariate regression analysis, the authors found that higher age and use of high dose vitamin C were both independently associated with increased use of vasopressors. The incidence of acute kidney injury was significantly higher in the PRESSOR group compared to the NoPRESSOR group, but there was no independent association between vasopressor use and mortality.

The study by Pape et al comprised 111 patients divided into patients receiving fluid and vasopressor (VP+) (*n* = 20) versus only fluid (VP−) (*n* = 91).^21^ Study results revealed that patients in the VP+ group were older, had a higher %TBSA burnt, higher fluid requirements in the first 24 h, and higher Baux scores compared to the VP− group. VP+ patients required dialysis more frequently and had a higher mortality rate compared to VP− patients.

### 3.3 Assessment of study quality

Overall study quality, assessed according to the Newcastle‐Ottawa scale, differed in the two studies.^20^ In the Pape study, the overall study quality was uncertain due to limited description of study methods. On the contrary, the Adibfar study was given the highest score in every quality assessment domain (Table 3). Both studies consisted of ICU burn patients with adequate assessment of interventions and outcomes. Only the Adibfar study controlled for confounding factors. The length and adequacy of follow‐up was uncertain in the Pape study and considered good in the Adibfar study.

**TABLE 3:** Study quality assessment according to the Newcastle‐Ottawa scale^20^

Quality assessment domain	Adibfar et al^22^	Pape et al^21^
**Representativeness**		
A: Truly representative	A	A
B: Somewhat representative		
C: Selected group		
D: No description of the derivation of the cohort		
**Selection of nonexposed**		
A: Drawn from same community as the exposed	A	A
B: Drawn from a different source		
C: No description of derivation of nonexposed		
**Ascertainment of exposure**		
A: Secure record	A	A
B: Structured interview		
C: Written self‐report		
D: No description		
**Incident disease**		
A: The outcome was not present at start of study	A	A
B: The outcome may be present at start of study		
**Comparability**		
A: Controls for demographics/comorbidities	A	
B: Controls for any additional factor (e.g., age)		
C: Not done		C
**Assessment of outcome**		
A: Independent/blind assessment	A	A
B: Record linkage		
C: Self‐report		
D: description		
**Length of follow‐up**		
A: Long enough for outcomes to occur	A	
B: Might not be long enough for outcomes to occur		B
**Adequacy of follow‐up**		
A: Complete follow‐up	A	
B: Subjects lost was unlikely to introduce bias		
C: Follow‐up rate 90% or lower		
D: No statement		D

### 3.4 Additional findings regarding use of vasoactive and/or inotropic drugs

Among studies not included there were publications that reported use of vasoactive and/or inotropic drugs in burn patients, but with a research question out of the scope of this systematic review.^23^, ^24^, ^25^ Among these, several studies discussed the use of vasoconstrictor drugs in burn patients as a local tumescent injection to limit blood loss during burn surgery, considered irrelevant to resuscitation.^26^, ^27^, ^28^, ^29^, ^30^, ^31^ Yet, some publications reported treatment with vasopressor and fluids in both treatment groups, but studied the additional use of other drugs, like hydrocortisone, in one of the groups.^25^

## 4 DISCUSSION

This systematic review revealed that there is lack of evidence on how often vasoactive and/or inotropic drugs are added to fluids during initial resuscitation of burn patients, and benefits and harms of adding such drugs. The present systematic review identified two small retrospective studies comparing early fluid resuscitation with and without the addition of intravenous vasopressors. The study of Adibfar et al revealed that vasopressors were used in 31% of the patients and that advanced age was the main predictor of vasopressor use during burn resuscitation.^22^ The patients who received vasopressors also had significantly more extensive and deeper burns and increased need of mechanical ventilation. These results were in line with findings by Pape et al, who found that high Baux score and high %TBSA burnt were associated with vasopressor use.^21^ In the latter study, vasopressors were used in 18% of the patients, and vasopressor use was associated with more frequent dialysis treatment and increased mortality.^21^

Clinicians treating patients with major burns resuscitate with crystalloids to avoid hypovolemia and ensure tissue perfusion. In patients who remain hypotensive despite adequate volume therapy, advanced hemodynamic monitoring may help to assess whether the patient has a hypovolaemic shock, or if there are elements of other types of shock such as cardiogenic and/or septic shock. However, in patients who do not respond adequately to volume therapy, there are in fact few alternatives to vasoactive and/or inotropic drugs.

Initial fluid resuscitation of patients with major burns is challenging and there is a tight balance between burn shock and fluid overload.^15^, ^32^, ^33^ Reported strategies for fluid resuscitation vary widely, especially concerning the amount of fluid administered.^34^ Guidelines typically promote consensus formulae of 2–4 ml/kg/%TBSA burnt of crystalloid fluid administered during the first 24 h after injury.^6^, ^8^, ^12^ Vasoactive and/or inotropic drugs might benefit the patient in the acute phase of resuscitation by increasing cardiac output and/or vascular resistance, and thereby improving tissue perfusion and reducing the risk of oedema due to over resuscitation.^17^, ^35^ However, the potential constraining effects on the peripheral microcirculation of injured skin may raise concerns on their applicability.^17^, ^36^ The systemic effects of vasoactive drugs are quite well recognized, but vasopressors' local impact on injured skin in burn patients is poorly evaluated. Knabl et al revealed that a temporary reduction in skin perfusion due to systemic epinephrine administration seemed to indicate progression of burn necrosis in rabbit models.^35^

Currently, use of vasoactive and/or inotropic drugs is not recommended in burn resuscitation guidelines.^6^, ^8^, ^12^ A recently published survey by Soussi et al revealed that 80% of intensivists considered norepinephrine in the initial resuscitation of burn patients as a strategy to reduce fluid requirements.^36^ This demonstrates that there is a considerable discrepancy between guidelines and real‐life considering the use of vasopressors in acute burn resuscitation.^6^, ^8^, ^12^ No burn guidelines have actively recommended against the use of vasoactive and/or inotropic drugs. The only available clinical guideline for how to apply vasoactive and/or inotropic drugs during resuscitation is published nonpeer reviewed by the European Burn Association. In their “European practice guidelines for burn care” they recommend adding vasopressors in the case of life‐threatening hypotension despite adequate fluid resuscitation, and inotropes if tissue hypoperfusion persists despite adequate fluid resuscitation and vasopressor administration.^37^

This systematic review has apparent limitations, the most important is that no published randomized controlled trials were identified from our literature search. Four publications identified by the literature search had to be excluded because they were not available. These were old publications that, based on their titles, were unlikely to fulfill the inclusion criteria. Additionally, we may have missed publications due to the language limitation of our literature search. With only two available studies, we were unable to perform meta‐analyses and to conclude on our research question. The quality of evidence is poor since less than 200 patients were compared, and the statistical strength of the results is limited. The included studies were two relatively small nonrandomized studies, whereof one published as abstract with uncertain quality.^20^ Results should be interpreted with caution since both studies were conducted as post hoc analysis without control and treatment groups during the patients' ICU stay. In both included studies, there were differences between the compared groups in patient age and full thickness % TBSA.

Still, the present study is strengthened by the systematic literature search performed and that the methods used to select studies and extract data were published before study start. Moreover, two independent collaborators independently screened studies for eligibility, evaluated quality, and extracted data according to preset criteria. The study design enables a presentation of all available evidence which is useful for clinical decision makers.

The implication of this systematic review for clinical practice is that clinicians lack evidence to decide whether vasoactive and/or inotropic drugs should be added to fluids during burn resuscitation or not. Multicenter studies comparing a restrictive and nonrestrictive protocol for vasoactive and/or ionotropic drugs during resuscitation are needed. Prospective studies within a population of major burns and with appropriate monitoring of cardiovascular and fluid status, burn depth, and adequate statistical power are mandatory to answer to what degree vasoactive and/or inotropic drugs should be part of resuscitation of patients with major burns.

In conclusion, this systematic review revealed that there is lack of evidence regarding benefits and harms of using vasoactive and/or inotropic drugs in addition to fluids during early resuscitation of major burn patients. Only two small retrospective studies were identified, and we certainly need more data to decide if vasoactive and/or inotropic drugs should be added to fluids or not. There seems to be a discrepancy between treatment guidelines suggesting use of fluid alone, and clinical practice studied in surveys indicating that vasoactive and/or inotropic drugs are frequently used.

## AUTHOR CONTRIBUTIONS

All listed authors have contributed to the manuscript substantially and have agreed to the final submitted version.

## FUNDING INFORMATION

This research did not receive any specific grant from funding agencies in the public, commercial, or not‐for‐profit sectors.

## CONFLICT OF INTEREST

None of the authors of this article have any conflict of interest in the manuscript, including financial, consultant, institutional, or other relationships that might lead to bias.

## TRIAL REGISTRATION

PROSPERO (CRD42019120317), registered 20 February 2019.

## Supporting information


**Appendix S1** Supporting Information.
