# Supplementary material for: Vasoactive and/or inotropic drugs in initial resuscitation of burn injuries: A systematic review
Source: Acta Anaesthesiol Scand. 2022 Jun 16;66(7):795–802. doi: 10.1111/aas.14095 (PMC9543770; doi:10.1111/aas.14095)
Supplement: Supplementary file 1 — Appendix S1 Supporting Information. [file AAS-66-795-s001.zip › AAS_14095_Appendix B Acta (revised) .docx]

**Appendix B: Literature search strategy**

Search date: 3. December 2021

We have searched the following electronic databases to identify relevant studies, number of hits in parentheses:

- PubMed (577)
- Embase (588)
- Cochrane Database of Systematic Reviews (3)
- Cochrane Central Register of Controlled Trials (143)
- SveMed+ (1)

Total number of hits: 1312

Number of hits after removal of duplicates: **1058**

**PubMed**

("Dobutamine"[Mesh] OR dobutamin*[Title/Abstract] OR "Dopamine"[Mesh:NoExp] OR dopamin*[Title/Abstract] OR "Epinephrine"[Mesh:NoExp] OR epinephrine*[Title/Abstract] OR adrenalin*[Title/Abstract] OR "Norepinephrine"[Mesh] OR "Droxidopa"[Mesh] OR "Normetanephrine"[Mesh] OR noradrenalin*[Title/Abstract] OR nor-adrenalin*[Title/Abstract] OR norepinephrin*[Title/Abstract] OR nor-epinephrin*[Title/Abstract] OR droxidopa[Title/Abstract] OR normetanephrin*[Title/Abstract] OR normetadrenalin*[Title/Abstract] OR "Vasoconstrictor Agents"[Mesh:NoExp] OR vasoconstrictor*[Title] OR vasopressor*[Title] OR "Cardiotonic Agents"[Mesh] OR cardiotonic[Title] OR inotrop*[Title]) **AND** ("Burns"[Mesh] OR "Burn Units"[Mesh] OR burn*[Title/Abstract] OR "thermal injury"[Title/Abstract] OR "thermal injuries"[Title/Abstract] OR scald*[Title/Abstract]) AND (Danish[lang] OR English[lang] OR Norwegian[lang] OR Swedish[lang])

**Embase** (1947 to current)

1 dobutamine/

2 dopamine/

3 epinephrine/

4 noradrenalin/

5 droxidopa/

6 normetadrenalin/

7 *vasoconstrictor agent/

8 *cardiotonic agent/

9 (dobutamin* or dopamin* or epinephrine* or adrenalin* or noradrenalin* or nor-adrenalin* or norepinephrin* or nor-epinephrin* or droxidopa or normetanephrin* or normetadrenalin*).ti.

10 (vasoconstrictor* or vasopressor* or cardiotonic or inotrop*).ti.

11 exp Burn/

12 Burn Patient/

13 (burn* or thermal injury or thermal injuries or scald*).ti.

14 or/1-10

15 or/11-13

16 14 and 15

17 limit 16 to (danish or english or norwegian or swedish)

**Cochrane Database of Systematic Reviews**

((dobutamin* OR dopamin* OR epinephrin* OR adrenalin* OR noradrenalin* OR nor-adrenalin* OR norepinephrin* OR nor-epinephrin* OR droxidopa OR normetanephrin* OR normetadrenalin* OR vasoconstrictor* OR vasopressor* OR cardiotonic OR inotrop*) AND (burn* OR (thermal NEAR injur*) OR scald*)): in Title Abstract Keyword

**Cochrane Central Register of Controlled Trials**

((dobutamin* OR dopamin* OR epinephrin* OR adrenalin* OR noradrenalin* OR nor-adrenalin* OR norepinephrin* OR nor-epinephrin* OR droxidopa OR normetanephrin* OR normetadrenalin* OR vasoconstrictor* OR vasopressor* OR cardiotonic OR inotrop*) AND (burn* OR (thermal NEAR injur*) OR scald*)): in Title Abstract Keyword

**SveMed+**

1 exp: "Dobutamine"

2 exp: "Dopamine"

3 exp: "Epinephrine"

4 exp: "Norepinephrine"

5 noexp: "Vasoconstrictor Agents"

6 noexp: "Cardiotonic Agents"

7 exp: exp:"Burns"

8 exp:"Burn Units"

9 1 OR 2 OR 3 OR 4 OR 5 OR 6

10 7 OR 8

11 9 AND 10
