# Supplementary material for: Vasoactive and/or inotropic drugs in initial resuscitation of burn injuries: A systematic review
Source: Acta Anaesthesiol Scand. 2022 Jun 16;66(7):795–802. doi: 10.1111/aas.14095 (PMC9543770; doi:10.1111/aas.14095)
Supplement: Supplementary file 1 — Appendix S1 Supporting Information. [file AAS-66-795-s001.zip › AAS_14095_Appendix C (Acta).docx]

**Appendix C: Study selection form**

**Reviewer, date:_____________________________________________________________________**

**Number, first author, year:_­­_________________________________________________________**

**Review summary:**

**Decision made by one reviewer (after fulfilling this study selection form)**

Include Ο (all questions below answered “yes”)

Discuss Ο (some question below answered “unknown”)

Exclude Ο (some question below answered “no”)

**Detailed review**

**1. Is it an empirical study or survey?**

Yes: Ο No: Ο Unknown: Ο

**2. Is the study reporting data on vasoactive and/or inotropic drugs in burn injury patients?**

Yes: Ο No: Ο Unknown: Ο

**3. Is the population human burn injury patients?**

Yes: Ο No: Ο Unknown: Ο

**4. Is the intervention use of vasoactive and/or inotropic drugs within the first 48 hours after burn injury?**

Yes: Ο No: Ο Unknown: Ο

**5. Is the comparator use of intravenous fluids alone (any type/volume without vasoactive and/or inotropic drugs)?**

Yes: Ο No: Ο Unknown: Ο

**6. Reason to exclude**

1. Burn injury patients Yes: Ο No: Ο Unknown: Ο

2. Vasoactive and/or inotropic drugs (<48 h) Yes: Ο No: Ο Unknown: Ο

3. Control with intravenous fluid alone Yes: Ο No: Ο Unknown: Ο

4. Humans Yes: Ο No: Ο Unknown: Ο

5. Study design: intervention/observation Yes: Ο No: Ο Unknown: Ο

6. Study design: survey or audit Yes: Ο No: Ο Unknown: Ο

7. Study design: case report Yes: Ο No: Ο Unknown: Ο

8. Study design: letter, comment of note Yes: Ο No: Ο Unknown: Ο

9. Study design: review or meta-analyse Yes: Ο No: Ο Unknown: Ο

10. Study design: consensus or guideline Yes: Ο No: Ο Unknown: Ο

11. Study design: editorial Yes: Ο No: Ο Unknown: Ο

12. Study design: quality improvement Yes: Ο No: Ο Unknown: Ο

13. Study design: study methodology Yes: Ο No: Ο Unknown: Ο

14. Study design: personal observation Yes: Ο No: Ο Unknown: Ο

15. Other reason: ___________________ Yes: Ο No: Ο Unknown: Ο

**Final decision made by the reviewers (after discussion with other reviewer(s))**

Include Ο (questions 1-5 above answered “yes”, 6-15 answered “no”)

Exclude Ο (some question above answered “no”), reason with the lowest number above:________
