# Supplementary material for: Vasoactive and/or inotropic drugs in initial resuscitation of burn injuries: A systematic review
Source: Acta Anaesthesiol Scand. 2022 Jun 16;66(7):795–802. doi: 10.1111/aas.14095 (PMC9543770; doi:10.1111/aas.14095)
Supplement: Supplementary file 1 — Appendix S1 Supporting Information. [file AAS-66-795-s001.zip › AAS_14095_Appendix D (Acta).docx]

**Appendix D: Data extraction form**

**Reviewer (name, date)**:______________________________________________________________

**Study number, from list:_____________________________________________________________**

**1. Study identification**

Study identifier (first author, year): __________________________________________________

Study location (country, city):_______________________________________________________

**2. Study method**

Study design:_____________________________________________________________________

**3. Study participants**

Number of patients: Total:________

With vasoactive and/or inotropic drugs:_______

Without vasoactive and/or inotropic drugs:________

Type of burn injuries:___________________________________________________________

Data collection period: ______________________________________________________________

Inclusion criteria:___________________________________________________________________

_________________________________________________________________________________

_________________________________________________________________________________

Exclusion criteria: __________________________________________________________________

_________________________________________________________________________________

_________________________________________________________________________________

TBSA: _________(%) Inhalation injury: Yes: Ο No: Ο Unknown: Ο

**4. Results**

| **Outcome reported** | **Dichotomous data** | | **Continuous data** | |
| --- | --- | --- | --- | --- |
| **Specify data** | **With vasoactive and/or inotropic drugs**  N sick /  N exposed | **Without vasoactive and/or inotropic drugs**  N sick /  N exposed | **With vasoactive and/or inotropic drugs**  Median / IQR  Mean / SD | **Without vasoactive and/or inotropic drugs**  Median / IQR  Mean / SD |
| Risk factor: Age (years) |  |  |  |  |
| Risk factor: Male gender (n=) |  |  |  |  |
| Risk factor: Body weight (kg) |  |  |  |  |
| Risk factor: Body height (cm) |  |  |  |  |
| Risk factor: Chronic kidney disease (n=) |  |  |  |  |
| Risk factor: Diabetes mellitus (n=) |  |  |  |  |
| Risk factor: Chronic hypertension (n=) |  |  |  |  |
| Risk factor: Coronary artery disease (n=) |  |  |  |  |
| Risk factor: TSBA  (%) |  |  |  |  |
| Risk factor: Inhalation injury (n=) |  |  |  |  |
| Risk factor: Intubated on arrival hospital (n=) |  |  |  |  |
| Total fluid infused on arrival hospital (ml) |  |  |  |  |
| Crystalloids infused on arrival hospital (ml), fluid type: |  |  |  |  |
| Colloids infused on arrival hospital (ml),  fluid type: |  |  |  |  |
| Fluid infused first 48 hours (ml). Time:_____ |  |  |  |  |
| Crystalloids infused on arrival hospital (ml),  fluid type: |  |  |  |  |
| Colloids infused on arrival hospital (ml),  fluid type: |  |  |  |  |
| Any other relevant variable (specify): |  |  |  |  |
| **Outcome reported** | **Dichotomous data** | | **Continuous data** | |
| **Specify data** | **With vasoactive and/or inotropic drugs**  N sick /  N exposed | **Without vasoactive and/or inotropic drugs**  N sick /  N exposed | **With vasoactive and/or inotropic drugs**  Median / IQR  Mean / SD | **Without vasoactive and/or inotropic drugs**  Median / IQR  Mean / SD |
| Time from burn to norepinephrine (hours from burn injury) |  |  |  |  |
| Average dose norepinephrine  (µg/kg/min) |  |  |  |  |
| Maximum dose norepinephrine  (µg/kg/min) |  |  |  |  |
| Time from burn to epinephrine (hours from burn injury) |  |  |  |  |
| Average dose epinephrine  (µg/kg/min) |  |  |  |  |
| Maximum dose epinephrine  (µg/kg/min) |  |  |  |  |
| Time from burn to dopamine (hours from burn injury) |  |  |  |  |
| Average dose dopamine  (µg/kg/min) |  |  |  |  |
| Maximum dose dopamine  (µg/kg/min) |  |  |  |  |
| Time from burn to dobutamine (hours from burn injury) |  |  |  |  |
| Average dose dobutamine  (µg/kg/min) |  |  |  |  |
| Maximum dose dobutamine  (µg/kg/min) |  |  |  |  |
| Lowest SBP first 48 h (mmHg) Time:_____ |  |  |  |  |
| Highest HR first 48 h (beats/min) Time:_____ |  |  |  |  |
| Shock first 48 h  (n=) |  |  |  |  |
| Arterial line first 48 hours (n=) |  |  |  |  |
| **Outcome reported** | **Dichotomous data** | | **Continuous data** | |
| **Specify data** | **With vasoactive and/or inotropic drugs**  N sick /  N exposed | **Without vasoactive and/or inotropic drugs**  N sick /  N exposed | **With vasoactive and/or inotropic drugs**  Median / IQR  Mean / SD | **Without vasoactive and/or inotropic drugs**  Median / IQR  Mean / SD |
| CVC first 48 hours  (n=) |  |  |  |  |
| PICCO first 48 hours (n=) |  |  |  |  |
| Echocardiography first 48 hours (n=) |  |  |  |  |
| Lowest CI first 48 hours (L/min/m^2^) |  |  |  |  |
| Lowest SVRI first 48 hours (dynx*s/cm5*m^2^) |  |  |  |  |
| Highest lactate first 48 hours (mmol/l) |  |  |  |  |
| Highest haemoglobin first 48 hours (g/L) |  |  |  |  |
| Bronchoscopy first 48 hours (n=) |  |  |  |  |
| Ventilator first 48 hours (n=) |  |  |  |  |
| Diuresis first 48 hours (ml) |  |  |  |  |
| Highest creatinine fist 48 hours (µmol/l) |  |  |  |  |
| Highest urea first 48 hours (mmol/L) |  |  |  |  |
| Renal replacement therapy (n=) |  |  |  |  |
| Surgery first 48 hours (n=) |  |  |  |  |
| Total number of surgical procedures (n=) |  |  |  |  |
| Total time on ventilator (days) |  |  |  |  |
| Total time on vasopressors (days) |  |  |  |  |
| Antibiotic treatment first 48 hours (n=) |  |  |  |  |
| SAPS II (score) |  |  |  |  |
| APACHE II (score) |  |  |  |  |
| Any other relevant variable (specify): |  |  |  |  |

| **Outcome reported** | **Dichotomous data** | | **Continuous data** | |
| --- | --- | --- | --- | --- |
| **Specify data** | **With vasoactive and/or inotropic drugs**  N sick /  N exposed | **Without vasoactive and/or inotropic drugs**  N sick /  N exposed | **With vasoactive and/or inotropic drugs**  Median / IQR  Mean / SD | **Without vasoactive and/or inotropic drugs**  Median / IQR  Mean / SD |
| ICU LOS (days) |  |  |  |  |
| Hospital LOS (days) |  |  |  |  |
| Mortality (n=),  time: |  |  |  |  |
| Mortality (n=),  time: |  |  |  |  |
| Mortality (n=),  time: |  |  |  |  |
| Health expenses,  Unit: |  |  |  |  |
| Other relevant parameter (specify):  ____________________ |  |  |  |  |
| Other relevant parameter (specify):  ____________________ |  |  |  |  |
| Other relevant parameter (specify):  ____________________ |  |  |  |  |
| Other relevant parameter (specify):  ____________________ |  |  |  |  |
| Other relevant parameter (specify):  ____________________ |  |  |  |  |
| Other relevant parameter (specify):  ____________________ |  |  |  |  |
